# Food Preferences of Patients with Citrin Deficiency

**DOI:** 10.3390/nu13093123

**Published:** 2021-09-06

**Authors:** Miki Okamoto, Yoshiyuki Okano, Mai Okano, Masahide Yazaki, Ayano Inui, Toshihiro Ohura, Kei Murayama, Yoriko Watanabe, Daisuke Tokuhara, Yasuhiro Takeshima

**Affiliations:** 1 Okano Children’s Clinic, Izumi 594-0071, Japan; comai_nu_un0710@yahoo.co.jp; 2 Department of Pediatrics, Hyogo College of Medicine, Nishinomiya 663-8501, Japan; ytake@hyo-med.ac.jp; 3 Department of Pediatrics, Kansai Medical University, Hirakata 573-1010, Japan; mai9ma11@yahoo.co.jp; 4 Department of Biological Sciences for Intractable Neurological Disorders, Institute for Biomedical Sciences, Shinshu University, Nagano 390-8621, Japan; mayazaki@shinshu-u.ac.jp; 5 Department of Pediatric Hepatology and Gastroenterology, Saiseikai Yokohamashi Tobu Hospital, Yokohama 230-0012, Japan; inui19@yacht.ocn.ne.jp; 6 Division of Clinical Laboratory, Sendai City Hospital, Sendai 982-8502, Japan; tohura@med.tohoku.ac.jp; 7 Department of Metabolism, Chiba Children’s Hospital, Chiba 266-0007, Japan; kmuraya@mri.biglobe.ne.jp; 8 Research Institute of Medical Mass Spectrometry, Kurume University School of Medicine, Kurume 830-0011, Japan; york@med.kurume-u.ac.jp; 9 Department of Pediatrics and Child Health, Kurume University School of Medicine, Kurume 830-0011, Japan; 10 Department of Pediatrics, Osaka City University Hospital, Osaka 545-0051, Japan; m1155519@med.osaka-cu.ac.jp

**Keywords:** adult-onset type II citrullinemia, aspartate glutamate carrier, carbohydrate toxicity, citrin, citrullinemia, CTLN2, diet therapy, food preference, SLC25A13

## Abstract

Citrin deficiency is characterized by a wide range of symptoms from infancy through adulthood and presents a distinct preference for a diet composed of high protein, high fat, and low carbohydrate. The present study elucidates the important criteria by patients with citrin deficiency for food selection through detailed analysis of their food preferences. The survey was conducted in 70 citrin-deficient patients aged 2–63 years and 55 control subjects aged 2–74 years and inquired about their preference for 435 food items using a scale of 1–4 (the higher, the more favored). The results showed that the foods marked as “dislike” accounted for 36.5% in the patient group, significantly higher than the 16.0% in the controls. The results also showed that patients clearly disliked foods with 20–24 (% of energy) or less protein, 45–54% (of energy) or less fat, and 30–39% (of energy) or more carbohydrate. Multiple regression analysis showed carbohydrates had the strongest influence on patients’ food preference (β = −0.503). It also showed female patients had a stronger aversion to foods with high carbohydrates than males. The protein, fat, and carbohydrate energy ratio (PFC) of highly favored foods among patients was almost the same as the average PFC ratio of their daily diet (protein 20–22: fat 47–51: carbohydrates 28–32). The data strongly suggest that from early infancy, patients start aspiring to a nutritional balance that can compensate for the metabolism dissonance caused by citrin deficiency in every food.

## 1. Introduction

Citrin deficiency is caused by variants in the SLC25A13 gene, which encodes the aspartate glutamate carrier protein in the mitochondrial inner membrane of hepatic, renal, and cardiac cells [1,2,3]. The aspartate glutamate carrier protein supplies aspartate generated in the mitochondria to the cytosol, constructs the malate-aspartate shuttle, and transports reduced nicotinamide adenine dinucleotide equivalent in the cytosol to the mitochondria. The loss of citrin function impairs glycolysis, gluconeogenesis from lactic acid, and synthesis of urea, protein, and nucleic acid as well as lipid metabolism and energy metabolism [2,3,4,5,6].

Citrin deficiency affects various metabolic pathways and may express different phenotypes at different life stages with a wide range of symptoms [5]. Neonatal intrahepatic cholestasis caused by citrin deficiency (NICCD) may be triggered in early life. The common clinical features of NICCD are jaundice, failure to thrive, and multiple amino acidemias, such as citrullinemia. Most of these conditions resolve between 6 and 12 months of age following treatment with medium-chain triglyceride (MCT) milk and lipid-soluble vitamins [7,8,9,10,11,12]. NICCD recovery is followed by an apparently healthy period called the adaptation/compensation period. Despite the appearance, evidence suggests that various clinical symptoms, such as hypoglycemia, fatigue, failure to thrive, and fatty liver, are actually present during this period, which can affect the patients’ quality of life (QOL) [4,11,13], let alone the strong food preference. The condition could progress to pre-adult-onset type II citrullinemia (CTLN2), including serious fatigue, weight loss, loss of appetite, psychiatric symptoms, hyperammonemia, and citrullinemia [4,11,14,15]. Failure to thrive with dyslipidemia caused by citrin deficiency is also reported to be present during this period [16]. Therefore, it is important for patients in the adaptation/compensation stage to maintain physical wellness to prevent the onset of CTLN2 [11,13]. CTLN2 may become clinically apparent after adolescence and is known to present with hyperammonemia, citrullinemia, liver dysfunction, fatty liver, and neuropsychiatric impairments, such as disorientation, delirium, mental derangement, and episodes of sudden unconsciousness [3,4]. The current estimated number of CTLN2 patients is about 10% of the patients with citrin deficiency [4]. Liver transplantation was previously thought to be the only remedy for CTLN2, but a diet with high protein, high fat, and low carbohydrate along with medications has become the main treatment in recent years [17,18,19,20,21].

The unique diet of patients with citrin deficiency has been considered to play an important role in treatment since a nutritional survey conducted in 2008 on citrin deficient patients in the adaptation/compensation period [22]. It has been reported that their energy intake was 89% of that of the Japanese general population, with a protein: fat: carbohydrate (PFC) energy ratio of 20:40–50:30–37, compared with 15:30:55 in the general population [23]. This indicates lower energy, higher protein, higher fat, and lower carbohydrate in patients’ diets. This particular diet is presumed to be an adaptive behavior of patients to compensate for the metabolic dysfunction caused by citrin deficiency [24]. In response to an active nutritional intervention, which followed the above survey, led to a notable change in patients’ diet with an increase in energy intake to 115% of that of the general population with a shift in the PFC ratio of 20–22:47–51:28–32 [24]. Today, appropriate diet treatment is considered to prevent the onset of CTLN2 and help the wellness of children by preventing hypoglycemia, reducing fatigue, and improving QOL.

Based on knowledge gained from the past nutritional evaluation on energy intake and the ratio of three major macronutrients [22,24,25], the most effective dietary intervention for patients with citrin deficiency is not to limit food choices but to have patients chose foods without any dietary restriction. The present study was designed to understand the actual food choices of patients according to their preference, and to elucidate the important criteria for food selection. Towards this aim, we conducted a survey in patients and controls living in the same household and asked questions on their food preferences.

## 2. Materials and Methods

### 2.1. Study Design and Subjects

A survey inquiring about food preference, diagnosis, clinical phenotype at the time of diagnosis, physical data, and current treatment was distributed to patients with citrin deficiency through six large medical institutions and the Patient Association of Citrullinemia in Japan.

Seventy patients (40 males, 30 females) aged 2–63 years (mean ± SD, 18 ± 17 years) from 61 families diagnosed with citrin deficiency by genetic analysis and 55 control subjects (23 males, 32 females) aged 2–74 years (mean ± SD, 26 ± 19 years) enrolled in this study. Family members living in the same household served as the control subjects. The selection of the control group from the same household was based on the premise that evaluation of the diet under the same household allows the elimination of various potentially biased factors arising from regional or familial characteristics of food culture. Of the 49 patients aged 2–16 years, 45 had been diagnosed with NICCD during infancy. The remaining 4 patients were diagnosed during the adaptation/compensation period. Out of 11 patients aged 17–39 years, four were diagnosed with NICCD during infancy, five during the adaptation/compensation period, and two were diagnosed with CTLN2 during adulthood. Among the 10 patients aged over 40 years old, nine were diagnosed with CTLN2 and one was in the adaptation/compensation period. None of the CTLN2 patients had undergone liver transplantation previously. All patients had received nutrition counseling, and some patients were being treated with MCT oil (n = 13), sodium pyruvate (n = 10), or arginine (n = 11).

### 2.2. Survey

The survey was conducted among patients with citrin deficiency and controls on likes/dislikes using a scale of 1–4 (1: dislike very much, 2: dislike, 3: like, 4: like very much) and n/a: never had it before on 435 specific food items under the following 13 food categories; grains (65 items), potatoes (21 items), beans (17 items), nuts (5 items), fruits (26 items), mushrooms (8 items), seaweeds (11 items), vegetables (71 items), fish (49 items), eggs (10 items), meat (50 items), dairy products (17 items), and confectioneries/beverages (85 items). Each food item was listed in the name of the served form of a dish to capture the preferences according to the seasonings and cooking methods, with the exception of fruits, vegetables, fish, and meat categories, where the ingredient itself was also included to determine preferences in kinds and parts of it (Supplementary Materials Table S1). The food preferences of young children were filled out with the help of their guardians.

### 2.3. Protein, Fat, Carbohydrate (PFC) Energy Ratio in Food

The nutritional component for 435 food items was calculated using the Standard Tables of Food Composition in Japan 2015 [26]. The PFC ratio was determined with the ratio of calories from protein, fat, and carbohydrate from the total calories in the food. The cooking data and recipes listed in Microsoft Excel add-in software Excel Eiyokun^®^ ver.8 (Kenpakusha, 2016) were used to define the content and amount of ingredients.

### 2.4. Statistical Analysis

Food preference data were calculated as the percentage of patients and controls with ratings of 1,2,3, and 4 for each of 435 food items (preference score). Differences between the distribution ratios of the four degrees of food preference were examined for statistical significance using the χ2 test of independence, and residual analysis was performed if independence was not observed. The average value of the scores of patients or controls for each food was used to define the food preference score. The 435 food items were classified into 13 food categories, and the average value of the food preference scores for each food category was regarded as the category preference score. All data are expressed as the mean ± standard deviation. The scores were compared by the Student’s *t*-test if the f-test found homoscedasticity of variances, and by Welch’s *t*-test if the f-test did not find homoscedasticity of variances. The Pearson product-moment correlation coefficient was used to analyze the correlation between the preference score and the PFC ratio of the food. The forward-backward stepwise selection method was used for multiple regression analysis. The PFC ratio is the energy composition ratio of protein, fat, and carbohydrate to the total amount of energy. The sum of protein, fat, and carbohydrate ratios makes 100% of energy. Therefore, there are correlations between the independent variables, and it creates multicollinearity. The multicollinearity may offset the effect of each independent variable on the dependent variable and interfere with the proper analysis. We used the weight of protein, fat, and carbohydrate per 100 g of food instead of the PFC ratio in the multiple regression analysis to avoid the risk. The forward-backward stepwise selection method was used for multiple regression analysis. Multiple regression analysis was performed using Bell Curve for Excel version 3.21 (Social Survey Research Information Co., Osaka, Japan), and for the others using Microsoft Excel 2010 version 14. A *p*-value less than 0.05 was considered significant.

## 3. Results

### 3.1. Comparison of Preference Scores between Patients and Controls

**Figure 1:**
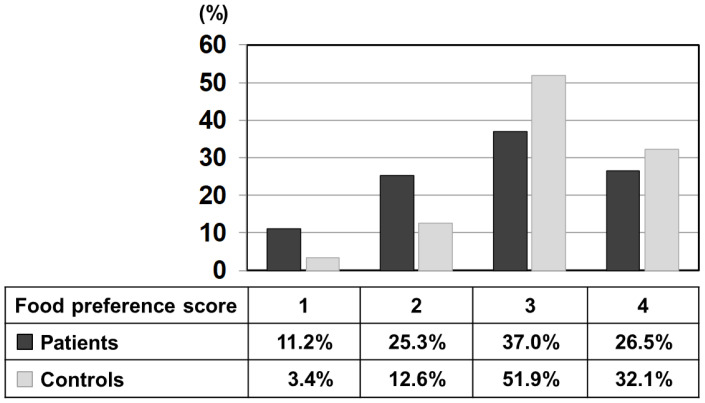
Relative frequency distribution of each preference score (1–4) of all listed 435 foods marked by patients and control subjects. Black bars: patients, grey bars: control subjects.

Figure 1 shows the percentage of each score selected by patients and controls using the relative frequency distribution for all foods. The mean value of the preference score (a scale of 1–4) of patients was 2.79 ± 0.96, which was significantly lower than that of the control (3.13 ± 0.75). To illustrate further, 3 points (like) and 4 points (like very much) accounted for 84% in the control, while the same scores accounted for 63.5% in the patients, significantly lower than the control. On the other hand, 1 point (dislike very much) and 2 points (dislike) accounted for 36.5% in patients, significantly higher than 16.0% in the control. In other words, this result indicates that the patient’s unique diet relates to the choice of dislikes.

### 3.2. Food Preferences and PFC Ratio of Food Categories

**Figure 2:**
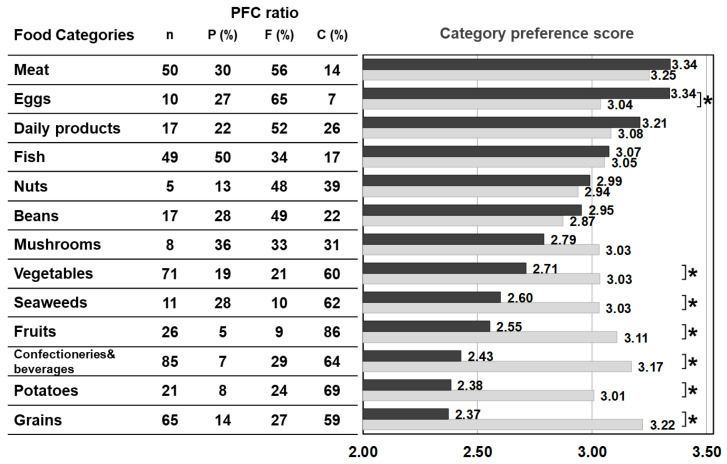
PFC ratio of the 13 food categories and the category preference score marked by patients with citrin deficiency and control subjects. Black bars: patients, grey bars: control subjects. * *p* < 0.05. The category preference score is the averaged preference score of foods within the same category, marked by patients and controls.

Figure 2 shows the average value of the food preference scores of food items that belong to the same category (‘category preference score’) and the averaged PFC score of each category. The category preference scores of the control were approximately 3 (mean 3.06 ± 0.10 points) across all categories, regardless of the PFC ratio. On the other hand, the category preference scores of patients varied and were dependent on the category. The category preference scores of meats, eggs, daily products, and fish which are high in protein, high in fat, and low in carbohydrate, were 3 or higher (3.24 ± 0.13), whereas the category preference scores of vegetables, seaweeds, fruits, confectioneries/beverages, potatoes, and grains, which contain 50% more high carbohydrate, were 2.51 ± 0.14, significantly lower compared with 3.10 ± 0.09 in the control group.

### 3.3. Food Preferences and PFC Ratio

**Figure 3:**
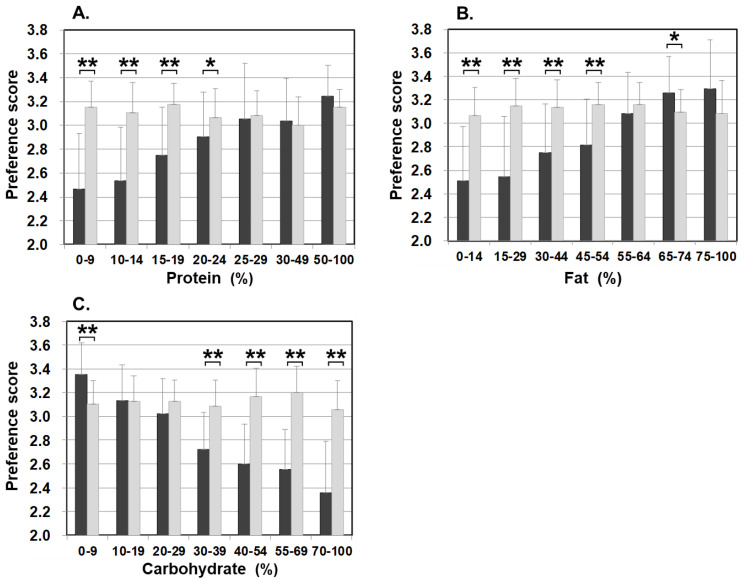
Food preference scores marked by patients and controls for foods broken down by the PFC ratio of each nutrient ((**A**) protein, (**B**) fat, and (**C**) carbohydrate). Black bars: patients, grey bars: control subjects. * *p* < 0.05, ** *p* < 0.001.

**Figure 4:**
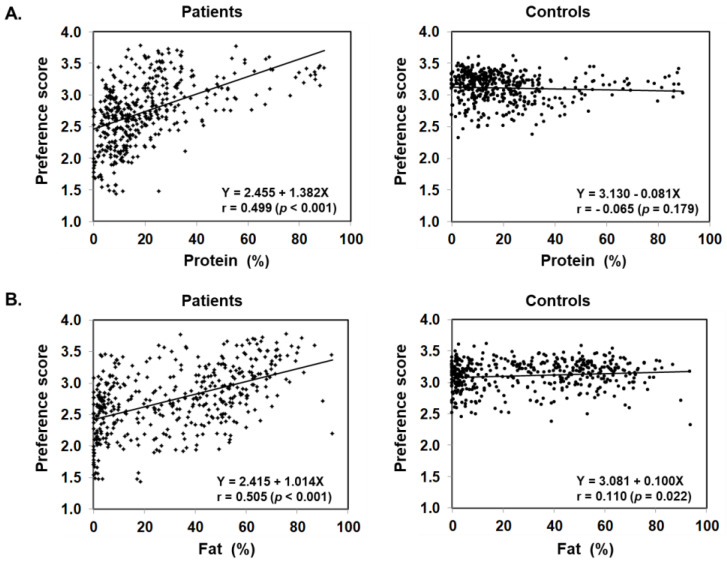

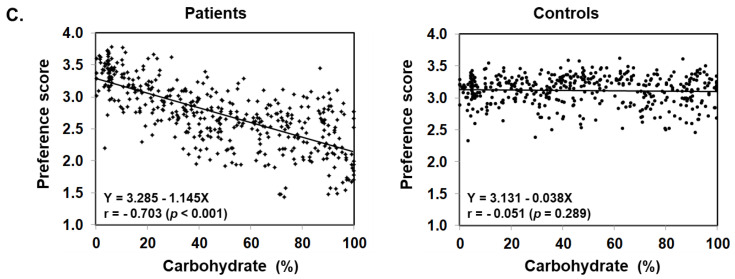
Scatter plot of protein (**A**), fat (**B**), and carbohydrate (**C**) content ratios based on the PFC ratio of 435 foods and food preference scores marked by patients with citrin deficiency and control subjects.

Figure 3 illustrates food preference according to the nutrition content of foods. In comparison to the control, patients scored significantly lower for foods containing 20–24% or less protein, 45–54% or less fat, and 30% or more carbohydrates. The nutrition content did not influence the preference score of the control. In addition, we also analyzed the correlation between the content ratios of the protein, fat, and carbohydrate in food and the preference score (Figure 4). For patients, the higher the protein content was, the higher the preference score became, showing a significant positive correlation (r = 0.499, *p* < 0.001). The same was true for fat, the higher the content was, the higher the preference score became, showing a significantly positive correlation (r = 0.505, *p* < 0.001). On the other hand, for carbohydrates, the higher the content was, the lower the preference score became, with a significantly strong negative correlation (r = −0.703, *p* < 0.001). No such correlation was found in the control.

### 3.4. Effects of Protein, Fat, and Carbohydrate on Food Preference in Patients

Multiple regression analysis was used to investigate the effects of the PFC ratio on the choice of food according to the preference and determine the most influential nutrient among the three nutrients to the patients. In this analysis, the weight of nutrition per 100 g of food was used instead of the PFC ratio of the content. The preference scores served as the dependent variables, and the weight of each of the protein, fat, and carbohydrate contents served as independent variables. The analysis showed that protein, fat, and carbohydrate were all selected as significant related factors (*p* < 0.001), with protein and fat found as promoters and carbohydrate as a suppressor of food preferences (Table 1). These nutrients explain 47.7% of the patients’ food preference and it was found that carbohydrate has the strongest influence on food preference (protein β = 0.288, fat β = 0.352, carbohydrate β = −0.503).

### 3.5. Food Preference in Relation to Age

**Figure 5:**
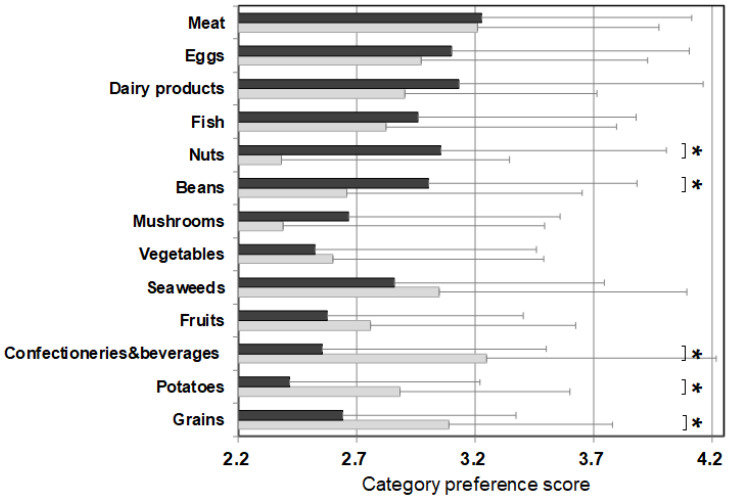
Category preference scores of 13 categories in 2–6-year-old patients and the age-matched control subjects. Black bars: patients, grey bars: control subjects. * *p* < 0.05.

The averaged food preference scores of patients and controls in five age groups are listed in Table 2. The average preference score of the patient group was significantly lower in all age groups, including the 2–6 years age group than that of the control group. Figure 5 shows the category preference scores of 2–6 years old patients and the control. The control group rated low for nuts, beans, mushrooms, and vegetables which are all commonly disfavored by small children [27,28,29]. Of the said categories, nuts and beans were marked significantly higher by patients compared to the control. On the other hand, confectioneries/beverages, potatoes, and grains were marked significantly lower by patients than the control in this age group. Patients of the 2–6 years old group rated their preference in a similar fashion as patients as a whole (Figure 2) rather than the age-matched control group. The results indicate that the patients’ sense of preference and aversion to carbohydrates are already determined as early as the age of 2–6 years (Figure 5).

The control group rated low for nuts, beans, mushrooms, and vegetables which are all commonly disfavored by small children.

### 3.6. Sex-Related Food Preferences

**Figure 6:**
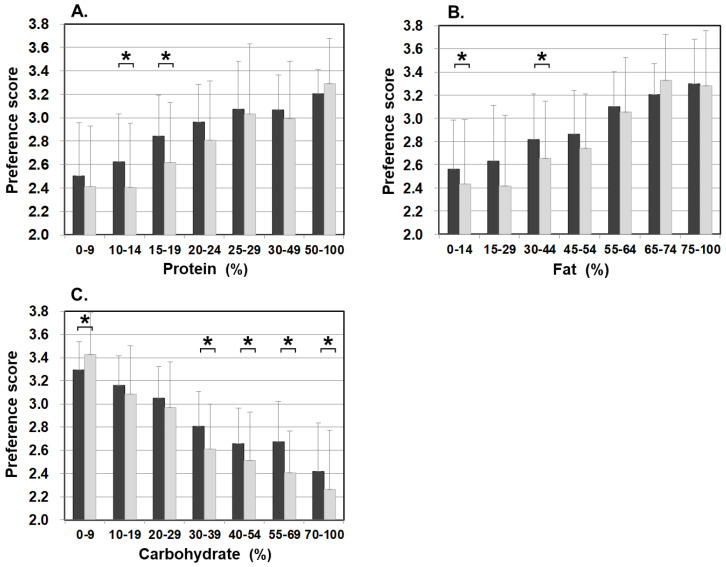
Food preference scores marked by male patients and female patients for foods broken down by the PFC ratio of each nutrient ((**A**) protein, (**B**) fat, and (**C**) carbohydrate). Black bars: male patients, grey bars: female patients. * *p* < 0.05.

The overall preference score of female patients was 2.74 ± 1.00, which was significantly lower than 2.82 ± 0.92 of their male counterparts. The female control also scored significantly lower (3.06 ± 0.75) than the male control (3.23 ± 0.74), which illustrates females, in general, are more selective and critical in food evaluation than males. The differences in scoring between female patients and female control were in the nutrient contents. Female patients showed varying preference scores depending on the protein, fat, and carbohydrate contents, whereas the female control showed the low score regardless (Figure 6). We also analyzed the sex-related differences in nutrient contents in the patients. For proteins, female patients scored significantly lower for food with lower content of protein of 10–14% and 15–19% than male patients, but there was no significant difference between the two sexes for food with 20% or higher protein content. For fat, female patients scored significantly lower for foods with a fat content of 0–14% and 30–44% than male patients, and there was no significant difference between the two sexes in other fat content. For carbohydrates, female patients scored significantly lower for preference for food with 30% or more carbohydrate content than male patients, and there was no significant difference between the two sexes in food with 10–29% carbohydrate content. These findings suggest that female patients have a stronger aversion to foods with low protein, low fat, and high carbohydrate contents than male patients.

### 3.7. Food Preference in Relation to Cooking Methods and Seasonings

**Figure 7:**
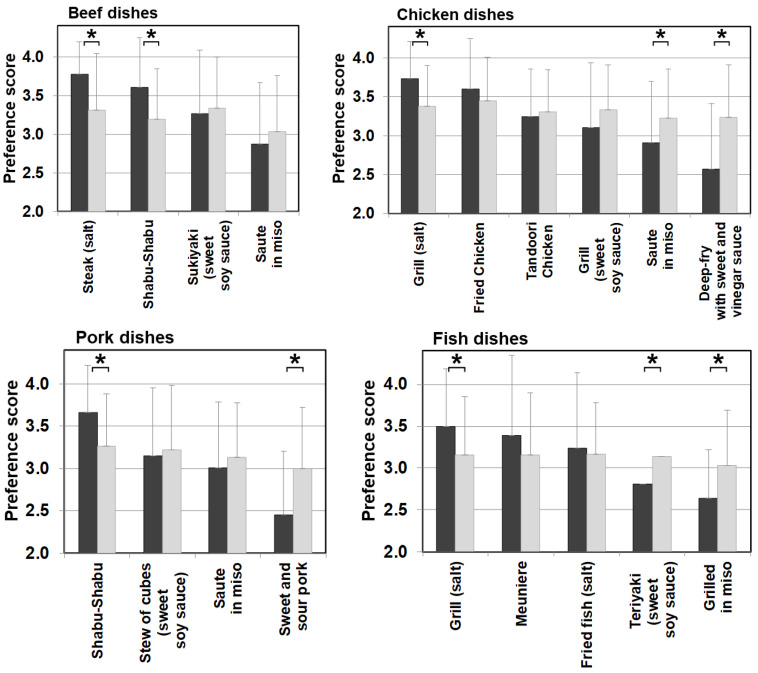
Food preference scores marked by patients and controls for foods broken down by cooking methods and seasonings of beef, chicken, pork, and fish dishes. Black bars: patients, grey bars: control subjects. * *p* < 0.05.

Differences in food preference were also noticed in cooking methods and seasonings in meat, eggs, dairy products, and fish categories, which are generally favorable among patients with citrin deficiency. Figure 7 shows meat and fish dishes as examples. Significantly higher scores for dishes with simple seasonings (i.e., salt and pepper) were observed in patients than in the control. On the other hand, sweeter dishes with sugar and miso used as ingredients had significantly lower preference scores in patients than the control when such ingredients did not have much impact on the PFC ratio of the dish. The same trend in preference according to seasonings was observed in other food categories, such as beans, mushrooms, vegetables, seaweeds, potatoes, and grains. No such trend was observed in the control group. This section may be divided into subheadings. It should provide a concise and precise description of the experimental results, their interpretation, as well as the experimental conclusions that can be drawn.

## 4. Discussion

Food preference is established through various factors, such as individual sensory ability, physiological and psychological characteristics, food attributes/images, and social environment including familial circumstances. Ultimately, foods that taste unlikeable may have adverse effects on the body, and choosing not to eat such foods is considered to be a self-defense behavior. Patients with citrin deficiency have long been known to have a strong food preference for high-protein and high-fat foods, such as meat, eggs, and dairy products with an aversion to sugar. Moreover, alcohol, excessive sugar intake, and certain medical practices, such as high glucose/high-calorie infusion and glycerol infusion, are also known to induce CTLN2 [30,31,32]. Patients in the adaptation/compensation period are involuntarily on a special diet to suppress the symptoms and maintain good QOL. It is very important to identify dietary patterns to support patients’ well-being and to conduct proper dietary counseling.

Since we conducted the survey in both patients and control subjects, we were able to study the characteristics of food choices and behavior of patients using the control for comparison (Figure 1). One of the striking findings of this study was that foods rated as ‘dislike’ by patients accounted for 36.5% of all foods, which is more than double that of the control group. The large number of foods marked in the survey as ‘dislike’ clearly depicts the defensive behavior in patients. In the food categories, scores for categories of potatoes, grains, confectioneries, fruits, seaweeds, and vegetables, which were mostly marked as ‘dislike’ by the patients, were significantly lower than those of the control when no differences between patients and controls were observed in the food categories of meat, eggs, daily products, and fish, which are mostly marked as ‘like’ by the patients (Figure 2). The survey results show that the food preference of patients is better characterized by food that they do not like, and the PFC ratio of such food is 5–28%:9–29%:59–86%, clearly containing a high energy portion of carbohydrate that the patients would definitely avoid.

The details of unfavorable foods of patients became evident after analyzing the food preference score and the PFC balance of the food. The study results showed that patients scored significantly lower than the controls on foods that contain 20–24% or less protein, 45–54% or less fat, and 30–39% or more carbohydrate. Conversely, patients scored higher (like/like very much) on foods that match the daily PFC ratio of 20–22%:47–51%:28–32% of patients’ diet in the adaptation/compensation period [24]. This finding means patients naturally chose foods that have the optimum nutritional balance for citrin deficiency. Another finding from the correlation and multiple regression analyses is that the carbohydrate amount contained in food has the strongest influence on patients’ preferences. Interestingly, however, citrin deficient patients showed a sensitive reaction to sweetness. The sweeter the food is, the lower the preference score is in patients, compared with the control, when the PFC ratio actually is not significantly affected by the sweet agents. Patients instead tend to favor a simple salty flavor (Figure 7).

Our findings suggest that child patients aged 2 to 6 years tend to disfavor high carbohydrate foods compared to the age-matched controls. It is said that children from one and a half years old are able to understand their favorite food, which makes them able to protect themselves and compensate for the metabolic dissonance from citrin deficiency by avoiding high carbohydrate foods from very early ages [32].

Our results showed that female subjects, in general, had lower preference scores and marked more food items as ‘dislike’ than the male subjects. It was also observed that the female controls equally rated lower in all foods regardless of the nutrient contents than male controls, whereas female patients clearly rated lower for low protein, low fat, and high carbohydrate foods, compared with the male patients. These findings could add support to a recent study that showed female patients take 1.8% more protein, 7% more fat, and 8.8% less carbohydrate in the PFC ratio than male patients [24], and perhaps explains the fewer cases of CTLN2 in females.

It is known that even a single occasion of excessive carbohydrate intake, similar to long-term high carbohydrate intake over time, can induce high ammonia levels and sickness in citrin deficiency [4,33]. In addition, the results of this survey clearly showed that patients dislike foods with 20–24% or less protein, 45–54% or less fat, and 30–39% or more carbohydrate. Since the PFC ratio of patients’ favored foods in this survey is almost the same as the reported PFC ratio obtained from the average nutritional evaluation of the patients’ diet, it is evident that patients aspire to a nutritional balance that can compensate for the metabolism dissonance caused by citrin deficiency in every food in every meal. Maintaining carbohydrate intake at a minimum level is key to achieving high QOL and asymptomatic conditions for patients during the adaptation/compensation period. It is also worth noting that the self-defense mechanism has already been established in infancy after NICCD recovery, by 2 years of age at the latest. These children naturally learn that high carbohydrate foods have an adverse effect on them, and they avoid such food almost as an instinct.

## Figures and Tables

**Table 1:** Three nutrients associated with food preference scores in patients with citrin deficiency shown in multiple regression analysis.

	Food Preference Score	
	β	r
Protein	0.288 *	0.479 *
Fat	0.352 *	0.328 *
Carbohydrate	−0.503 *	−0.452 *
R-squared	0.481 *	
Adjusted R-squared	0.478 *	

N = 435 foods * *p* < 0.05. The amount of protein, fat, and carbohydrates per 100 g of food was used.

**Table 2:** Food preference score by age in citrin deficiency patients and control subjects.

	Food Preference Score	
Age (Years)	Patients (n)	Control Subjects (n)
2–6	2.79 ± 0.92 (16)	2.94 ± 0.91 * (6)
7–11	2.76 ± 0.99 (17)	3.23 ± 0.88 * (14)
12–16	2.77 ± 0.96 (16)	3.35 ± 0.74 * (5)
17–39	2.75 ± 0.93 (11)	3.18 ± 0.67 * (13)
40–	2.91 ± 0.93 (10)	3.00 ± 0.61 * (17)

Data are the mean ± SD. * *p* < 0.05, between patients and controls of the same age group (by Student’s *t*-test and Welch’s *t*-test).

## Supplementary Materials

The following are available online at https://www.mdpi.com/article/10.3390/nu13093123/s1, Table S1: 435 foods with food categories.
