# Supplementary material for: Food Preferences of Patients with Citrin Deficiency
Source: Nutrients. 2021 Sep 6;13(9):3123. doi: 10.3390/nu13093123 (PMC8468903; doi:10.3390/nu13093123)
Supplement: Supplementary file 1 [file nutrients-13-03123-s001.zip › nutrients-1319340-supplementary.pdf]

Supplemental tableS1: 435 foods with food categories

Food Categories	n	
Meat	50	Beef loin, Beef fillet, Beef tongue, Minced beef and pork, Beef shabu-shabu with ponzu soy sauce, Beef shabu-shabu with sesame sauce, Beef shabu-shabu with homemade sauce, Sukiyaki of beef, Sauteed beef with balsamic vinegar, Sauteed beef with miso, Beef steak with salt, Beef steak with sauce, Japanese style hamburg steak with ponzu soy sauce, Hamburg steak with demi-glace sauce, Cutlet, Mince cutlet, Beef jerky, Corned beef, Beef stew, Pork loin, Pork fillet, Minced pork, Pork miso soup, Pork shabu-shabu, Stewed pork cubes with sweet soy sauce, Sauteed pork with soy sauce, Sauteed pork with miso, Gingered pork, Sweet and sour pork, Pork cutlet with ponzu soy sauce, Pork cutlet with sauce, Roast ham, Bacons, Dry-cured ham, Vienna sausage, Dried sausage, Chicken wing, Chicken breast meat, Chicken thigh, Chicken sashimi, Stewed chicken with ponzu soy sauce, Grilled chicken with salt and pepper, Grilled chicken with salt, Grilled chicken with sweet soy sauce, Sauteed chicken with miso, Tandoori chicken, Deep-fried chicken with salt, Deep-fried chicken with sweet vinegar sauce, Fried chicken, Smoked chicken sashimi
Eggs	10	Raw eggs, Egg tofu, Boiled egg, Fried eggs, Japanese style omelet, Omelet, Scrambled eggs, Deep-fried egg, Simmered quail egg, Smoked quail egg
Dairy products	17	Milk, Low fat milk, Unsweetened yoghurt, Sweetened yoghurt, Yoghurt with fresh cream, Yoghurt with fruits, Whipped cream, Mozzarella cheese, Camembert cheese, Shredded cheese, Shaved cheese, "6P cheese" (processed cheese), "Baby cheese" (processed cheese), Sliced cheese, Corn potage soup, Clam chowder, Cream stew
Fish	49	White fish (e.g. sea bream,pacific cod), Salmon, Red fish (e.g. tuna), Blue-backed fish (e.g. horse mackerel, sardine, mackerel, yellowtail, saury), Eel, Shishamo smelt, Tarako(salted cod roe), Ikura (salted salmon roe) , Crab, Squid, Octopus, Shrimp, Clam, Pacific oyster, Sea urchin, Hirakiboshi (salted and semi-dried fish), Shirasuboshi (boiled and dried whitebait), Mentaiko (salted cod roe with red hot pepper powder), Surume (dried squid), Tempura of squid, Dried scallop, Canned tuna, Canned boiled mackerel, Canned boiled mackerel with miso, Canned salmon, Oiled sardine, Kamaboko (fish-paste cake), Chikuwa (tube-shaped fish-paste cake), Satsuma age (deep-fried ball of fish paste), Hanpen(pounded fish cake), Fish sausage, Sashimi (raw sliced fish, shellfish or crustaceans), Sushi, Salted squid, Steamed fish with butter and soy sauce, Simmered fish with soy sauce, Stewed fish with sweet vinegar, Simmered fish with ume paste, Simmered fish in miso, Grilled fish with salt, Marinated fish, Fish teriyaki (baked fish with sweet soy sauce), Meuniere of fish, Grilled fish with miso, Deep-fried fish marinated in sweet vinegar, Fried fish with salt, Fried fish with ponzu soy sauce, Fried fish with worcestershire sauce, Fried fish with tartar sauce

Nuts	5	Almonds, Peanuts, Peanuts butter, Roasted chestnuts, Roasted ginkgo nut
Beans	17	Boiled Green soybeans, Simmered soybeans, Natto, Chilled firm tofu with soy sauce, Chilled silk tofu with soy sauce, Grilled tofu with miso, Simmered tofu with sweet soy sauce, Mapo tofu, Deep fried tofu with japanese soup stock, Baked deep-fried tofu, Simmered deep-fried tofu, Simmered deep-fried tofu with vegetables, Simmered freeze-dried tofu, Soy milk, Tofu skin, Deep fried tofu skin, Sauteed and simmered soy pulp
Mushrooms	8	Vinegared mushrooms, Sauteed mushrooms with soy sauce, Sauteed mushrooms with butter, Tempura of mushrooms, Clear soup of mushrooms, Miso soup of mushrooms, Consomme soup of mushrooms, Chinese soups of mushrooms
Vegetables	71	Pumpkin, Carrot, Broccoli, Japanese mustard spinach, Spinach, Tomatoes, Leek, Green bell pepper, Kidney beans, Green peas, Chinese chive, Asparagus, Cabbage, Cucumber, Edible burdock, Japanese radishes, Bamboo shoots, Onions, Eggplant, Chinese cabbage, Bean sprouts, Lettuce, Okra, Small sweet green pepper, Perilla, Green bok choy, East Indian lotus root, Sweet corn, Bitter melon, Garlic, Wasabi, Cucumber pickled in salty rice bran paste, Japanese radishes pickled with salty rice bran paste, Umeboshi (pickled and dried plum), Ginger in sweet vinegar, Kimchi（pickled chinese cabbage), Salad with japanese soy sauce dressing, Salad with italian dressing, Salad with french dressing, Salad with thousand Dressing, Salad with sesame dressing, Salad with caesar dressing, Salad with non-oil dressing, Salad with mayonnaise, Salad without dressing, Boiled spinach with soy sauce, Boiled spinach with sesame paste, Boiled spinach with peanuts butter, Boiled leek with vinegared miso, Cucumber with ume paste, Bean sprouts namuru, Vinegared cucumber, Simmered Japanese radish, Simmered pumpkin, Simmered vegetables with sweet soy sauce, Simmered chinese cabbage with miso, Braised Chinese cabbage with milk, Pumpkin soup, Grilled eggplant with salt, Grilled eggplant with ponzu soy sauce, Grilled eggplant with miso, Sauteed cabbage with salt and pepper, Sauteed cabbage with soy sauce, Sauteed cabbage with miso, Sauteed cabbage with worcestershire sauce, Sauteed eggplant with sweet vinegar, Sauteed vegetables with butter, Sauteed japanese mustard spinach with almond, Sauteed bean sprouts with curry, Tempura of vegetables with salt, Tempura of vegetables with tentsuyu broth
Seaweeds	11	Salted kombu, Simmered kombu with sweet soy sauce, Grilled nori seaweed, Seasoned nori seaweed, Simmered hijiki, Vinegared mozuku seaweed, Wakame stem, Vinegared wakame seaweed, Vinegared thick wakame leaves, Gelidium jelly with brown sugar syrup, Gelidium jelly with vinegared soy sauce
Fruits	26	Avocados, Strawberries, Oranges, Japanese persimmons, Kiwifruit, Grapefruit, Sweet cherries, Watermelon, Pears, Pineapple, Bananas, Grapes, Muskmelon, Peaches, Apples, Lemons, Yuzu, Strawberry jam, Orange marmalade, Blueberry

		jam, Mandarin oranges canned in syrup, Pineapple canned in syrup, Peaches canned in syrup, Fruits and peas canned in syrup, Olives in brine, Dried fruits,
Confectioneries& beverages	85	Shortcake, Chocolate cake, Baked cheesecake, Unbaked cheesecake, Fruit tart, Pancake, Apple pie, Cream puffs with custard cream, Cream puffs with fresh cream and custard cream, Plain doughnut, Chocolate doughnut, Sugar doughnut, "Pon de ring" (doughnuts made with tapioca starch), Roll cake, Baumkuchen, Cookies and Biscuits, "Soy joy" high protein type (baked goods made from soy flour with fruits and nuts), "Soy joy" high fat type (baked goods made from soy flour with fruits and nuts), "Soy joy" high carb type (baked goods made from soy flour with fruits and nuts), Bread with sweet bean paste, Bread with Jam, Bread with custard cream, Chocolate bread, Melon bread (sweet baked with a bread-dough interior and a cookie-dough exterior), Fried bread with soybean flour and sugar, Milk chocolate, Bitter chocolate, White chocolate, Almond chocolate, Peanut chocolate, "Pocky" (pretzels on a stick, coated with chocolate), "Kinokonoyama""Takenokonosato" (crackers or cookies coated with chocolate), "Koalanomarch" (chocolate wrapped in a cookie), Potato chips, "Jyagariko" (potato chips in the shape of a stick), "Kappaebisen" (snacks made with flour and shrimp), "Happyturn" (western-style rice crackers), Popcorn, "Pritz." (pretzels on a stick), "Ritz" (crackers), "Cheeza" (cheese flavored snacks), Coconut chips, Rice crackers with salt flavor, Rice crackers with soy sauce flavor, Rice crackers with curry flavor, Rice crackers with mayonnaise flavor, Karinto (deep-fried brown sugar snacks), Dango (sweet rice dumpling), Manjyu (japanese bun filled with sweet bean paste), Sweet bean jelly, Bracken-starch dumpling, Anko (sweet bean paste), Sugar flavored white kidney beans, Sweet chestnut, Mashed sweet potatoes with sweetened chestnuts, Ice cream (milk solids content of at least 15% and a milk fat content of at least 8%), Ice milk (milk solids content of at least 10% and a milk fat content of at least 3%), Lact ice (milk-solids content of 3% or greater), Soft serve ice cream, Sherbet, Shaved ice with flavored simple syrup, Pudding, Custard pudding, Fruit jelly, Coffee jelly, Dessert of powdered and jellied apricot kernels with fruit, Konnyaku jelly, Candy, Caramel, Soft candy, Gummi, Straight fruit juice, Fruit juice beverage, "Yakult" (lactic acid bacteria beverages), "Calpis" (lactic acid bacteria beverages), Cola (carbonated beverage), Clear soda (carbonated beverage), "Fanta" (carbonated beverage), "Oronamin C" (carbonated nutritional beverage), "Pocari sweat" (sports beverage), Black tea, Canned coffee with no sugar, Canned coffee with low sugar, Cafe ole canned coffee, Oral rehydration solution
Potatoes	21	Grilled konnyaku with miso, Dressed konnyaku with vinegared miso, Simmered konnyaku with japanese soup stock flavor, Simmered konnyaku with sweet soy sauce, Baked sweet potato, Tempura of sweet potato, Candied sweet potato, Dried sweet potato, Simmered taro in soy sauce, Simmered taro in miso, Boiled and fried potatoes, Mashed potato, Potato salad with mayonnaise, Fried Potatoes, Hashed potato, Potato croquettes, Vichyssoise, Chinese yam,

		Kudzu starch noodles with brown sugar syrup, Kudzu starch noodles with vinegared soy sauce, Sesame tofu
Grains	65	Meshi (cooked rice), Sekihan (steamed rice with adzuki beans or cowpeas), Gomoku meshi (rice seasoned and cooked with various ingredients), Rolled sushi (sushi made rolled in nori seaweed with a core of filling), Inari sushi (sushi wrapped in deep-fried tofu), Chinese-style fried rice, Rice omelet (omelet with a filling of ketchup-seasoned fried rice), Curry and rice from homemade, Curry and rice from retort or specialty stores, Zoni with clear soup (soup containing rice cakes and vegetables), Zoni with miso soup (soup containing rice cakes and vegetables),<br>Isobe mochi (rice cakes wrapped in nori), Abekawa mochi (rice cakes with soybean flour and sugar), Ohagi (rice ball coated with sweet bean paste), Toast with butter, Toast with jam, French toast, French bread, Bread roll, Croissants, Bread with fried noodles, Bread with croquette, Fried bread with curry, Hot dog, Hamburger, Teriyaki burger (marinated in sweet soy sauce and grilled), Fish burger, Pizza with tomato sauce, Pizza with white sauce, Pizza with curry sauce, Udon noodles in japanese soup stock, Udon noodles in miso, Udon noodles with meat, Udon noodles in curry, Somen noodles, Japanese buckwheat noodles, Spaghetti with mushrooms in Japanese soup stock, Spaghetti peperoncino, Spaghetti meat, Spaghetti carbonara, Spaghetti with spicy cod roe, Macaroni au gratin with white sauce, Macaroni salad with mayonnaise, Chilled Chinese noodles, Fried noodles with sauce, Salt flavored ramen noodles, Soy sauce flavored ramen noodles, Miso flavored ramen noodles, Pork bone flavored ramen noodles, Instant ramen noodles, Baked rice noodles with starchy sauce, Vermicelli noodle salad with chinese flavor, Vermicelli noodle salad with mayonnaise, Bean-paste vermicelli noodles, Takoyaki (octopus balls), Okonomiyaki (japanese pizza), Gyoza (grilled dumpling), Shumai (steamed dumpling), Chinese steamed buns with meat, Chinese steamed buns with curry, Chinese steamed buns with pizza, Chinese steamed buns with sweet bean paste, Meat pie, Cornflakes with milk, Cornflakes only
